# Evaluation of psychological distress, burnout and structural empowerment status of healthcare workers during the outbreak of coronavirus disease (COVID-19): a cross-sectional questionnaire-based study

**DOI:** 10.1186/s12888-023-05088-x

**Published:** 2024-01-22

**Authors:** Sara Taleb, Amir Vahedian-Azimi, Leila Karimi, Safa Salim, Farhan Mohammad, Dana Samhadaneh, Kalpana Singh, Nur-Run Hussein, Ali Ait Hssain

**Affiliations:** 1 Division of Genomics and Translational Biomedicine, College of Health and Life Science, Hamad Bin Khalifa University, Doha, Qatar; 2 Proteomics Core, Research department, Weill Cornell Medicine-Qatar, Doha, Qatar; 3 Trauma Research Center, Nursing Faculty, Baqiyatallah University of Medical Sciences, Tehran, Iran; 4 Behavioral Sciences Research Center, Life Style Institute, Nursing Faculty, Baqiyatallah University of Medical Sciences, Tehran, Iran; 5 Division of Biological and Biomedical Sciences, College of Health & Life Sciences, Hamad Bin Khalifa University, Doha, Qatar; 6 McGill University, Montreal, Canada; 7 Nursing Midwifery Research Department, Hamad Medical Corporation, Doha, Qatar; 8 KPJ Damansara Specialist Hospital, Petaling Jaya, Malaysia; 9 Medical Intensive Care Unit, Hamad General Hospital, Doha, Qatar; 10 Department of Medicine, Weill Cornell Medical College, Doha, Qatar; 11 College of Health and Life Science, Hamad Bin Khalifa University, Doha, Qatar

**Keywords:** COVID-19, Burnout, Depression, Anxiety, Stress, Structural empowerment, Predictors

## Abstract

**Background:**

In light of several recent studies, there is evidence that the coronavirus disease 2019 (COVID-19) pandemic has caused various mental health concerns in the general population, as well as among healthcare workers (HCWs). The main aim of this study was to assess the psychological distress, burnout and structural empowerment status of HCWs during the COVID-19 outbreak, and to evaluate its predictors.

**Methods:**

This multi-center, cross-sectional web-based questionnaire survey was conducted on HCWs during the outbreak of COVID-19 from August 2020 to January 2021. HCWs working in hospitals from 48 different countries were invited to participate in an online anonymous survey that investigated sociodemographic data, psychological distress, burnout and structural empowerment (SE) based on Depression Anxiety and Stress Scale 21 (DASS-21), Maslach Burnout Inventory (MBI) and Conditions for work effectiveness questionnaire (CWEQ_II), respectively. Predictors of the total scores of DASS-21, MBI and CWEQ-II were assessed using unadjusted and adjusted binary logistic regression analysis.

**Results:**

Out of the 1030 HCWs enrolled in this survey, all completed the sociodemographic section (response rate 100%) A total of 730 (70.9%) HCWs completed the DASS-21 questionnaire, 852 (82.6%) completed the MBI questionnaire, and 712 (69.1%) completed the CWEQ-II questionnaire. The results indicate that 360 out of 730 responders (49.3%) reported severe or extremely severe levels of stress, anxiety, and depression. Additionally, 422 out of 851 responders (49.6%) reported a high level of burnout, while 268 out of 712 responders (37.6%) reported a high level of structural empowerment based on the DASS-21, MBI, and CWEQ-II scales, respectively. In addition, the analysis showed that HCWs working in the COVID-19 areas experienced significantly higher symptoms of severe stress, anxiety, depression and higher levels of burnout compared to those working in other areas. The results also revealed that direct work with COVID-19 patients, lower work experience, and high workload during the outbreak of COVID-19 increase the risks of negative psychological consequences.

**Conclusion:**

Health professionals had high levels of burnout and psychological symptoms during the COVID-19 emergency. Monitoring and timely treatment of these conditions is needed**.**

**Supplementary Information:**

The online version contains supplementary material available at 10.1186/s12888-023-05088-x.

## Background

In December 2019, a new type of coronavirus disease called coronavirus disease of 2019 (COVID-19) was first detected in China, and it rapidly spread worldwide in the following six months [1]. The disease was caused by a highly contagious and novel coronavirus known as severe acute respiratory syndrome coronavirus 2 (SARS-CoV-2) [1]. The World Health Organization (WHO) declared the outbreak a global pandemic in March 2020 [https://www.who.int/docs/default-source/coronaviruse/mental-health-considerations.pdf] . In fact, the outbreak of COVID-19 has become an unprecedented international public health emergency, resulting in various health and psychological problems among the general population, including healthcare workers (HCWs) [2]. This disease not only raises public health concerns but also leads to several forms of psychological distress including; anxiety, stress, depression, burnout, irritability, insomnia, and posttraumatic stress disorder (PTSD) [3–6].

The global healthcare system has faced unprecedented challenges in combating the spread of SARS-CoV-2, with HCWs on the frontline [7, 8]. In the early stages of the pandemic, HCWs experienced inadequate training in infection control, a shortage of personal protective equipment (PPE), and an overwhelming workload [9, 10]. In addition, the direct exposure to COVID-19 patients increased their risk of infection, and the fear of transmitting the virus to their families made them susceptible to mental distress [11]. Studies based on COVID-19 have provided emerging evidence of the negative psychological impact on HCWs from different countries [12–15]. A meta-analysis, which includes 108,931 medical staff individuals from 69 articles across four different countries (China, Iran, Italy and Turkey), reported that the pooled prevalence rates of anxiety, depression, and insomnia were 37%, 34% and 39%, respectively, during the COVID-19 pandemic [16]. Another meta-analysis by Lee et al. [17], examined a total of 401 studies, involving 458,754 participants from 58 different countries, revealed that the prevalence of depression was 28.5%, anxiety was 28.7%, PTSD was 25.5% and insomnia was 24.4%. Pappa et al. [18], has conducted a meta-analysis on 13 studies with 33,062 participants mainly from China, which reported the pooled prevalence of anxiety, depression and insomnia were 23.2%, 22.8% and 38.9%, respectively. A cross-sectional study on HCWs in Egypt and Saudi Arabia indicated 69%, 58.9%, 55.9% and 37.3% had depression, anxiety, stress, and insomnia, respectively [19]. A cross-sectional survey from Qatar reported that 71.4% of physician and 74.4% of nurses of intensive care unit (ICU) experienced moderate-to-severe perceived stress and high PTSD symptoms among them who works directly with COVID-19 patients [20]. Moreover, a national study from Qatar showed the high-risk perception and psychological impact of COVID-19 pandemic among HCWs in healthcare settings [21].

Undoubtedly, there is a need to understand the psychological distress experienced by HCWs who are directly or indirectly involved in the treatment and care of COVID-19 patients. This understanding will help identify and address risk factors for adverse mental health outcomes among HCWs and provide necessary interventions. Therefore, we conducted a multi-center, cross-sectional, exploratory web-based survey aimed at assessing the psychological distress, burnout, and structural empowerment status of HCWs from various regions worldwide (48 countries), with the majority of our participants being from Qatar.

## Methods

### Study design and ethical approval

This multi-center, cross-sectional web-based questionnaire survey was conducted on HCWs from 48 different countries, with the majority of them being from Qatar between August 2020 and January 2021. The study protocol was reviewed and approved by the Hamad Medical Corporation Institutional Review Board (MRC–05–006), and all study participants signed an electronic informed consent within the survey. The study was performed in accordance with the Declaration of Helsinki of the World Medical Association [22], and the recommendations of the Strengthening the Reporting of Observational Studies in Epidemiology (STROBE) statement [23].

### Participants

The eligible participants from different countries were contacted via email invitation, which include an information letter and a link to our questionnaires. These questionnaires were developed using the survey monkey tool (https://www.surveymonkey.com). The information letter provided a clear explanation of the aim of our study and the survey format to the invited HCWs. It also extended an invitation for them to participate voluntarily in the study, assuring them that their identity would remain anonymous and their information would be kept confidential. Our inclusion criteria were as follows: (a) HCWs included physicians, nurses, therapists and others (paramedics, surgeon, anesthesiologist and dietitian); (b) aged ≥ 18 years old, and (c) currently working in a hospital managing patients infected or potentially infected with COVID-19. HCWs who reported working in academic and research setting but not in hospitals managing COVID-19 were excluded. Due to the low response rate, a reminder email was sent to the invited HCWs. The Multiple Responses option in the survey monkey was disabled to prevent duplicate responses. Participants were allowed to terminate or continue with the survey at the end of each section.

### Data collection

The research data was collected with a four-part measuring tool; (a) sociodemographic characteristics of the participants and other COVID-9 related background data were collected using a self-developed questionnaire; (b) depression, anxiety, and stress status of participants were collected using the Depression, Anxiety and Stress Scale-21 (DASS-21) scale [24]; (c) burnout levels were measured by the Maslach Burnout Inventory (MBI) questionnaire [25]; and (d) structural empowerment status was collected by the Conditions for Work Effectiveness (CWEQ-II) questionnaire [26]. Study questionnaires are availablein Supplementary File 1.

Sociodemographic data consisted of gender, age, marital status, having children, job position, professional title, education levels, work experience, working hours per week, working in an area with COVID-19 patients, directly working with COVID-19 patients, work experience in contact with COVID-19 patients, receiving specific training for COVID-19, working hours per week during the outbreak COVID-19, taking care of COVID-19 patients in the last 24 h., last time caring for COVID-19 patients, history of mental illness, current use of medication for mental illness, and family history of mental illness.

### Research instruments

- DASS-21: This questionnaire was designed and validated by Lovibond in 1995 to measure the psychological distress in a community with 21 items [24]. DASS-21 is a unique, simple, and approved instrument for assessing depression, anxiety, and stress both in both clinical settings and communities [27]. DASS is a short screening tool that measures depression, anxiety, and stress through a 21-item self-report questionnaire. For each disorder, seven questions are considered, and the final score is obtained by totaling the scores of the questions related to it. Each question is scored using a Likert-scale ranging from 0 (did not apply to me at all/never) to 3 (applied to me very much, or most of the time/almost always). Higher scores indicated a higher level of disorder based on a specific classification scoring system. Individuals were categorized into normal, mild, moderate, severe, and extremely severe based on their responses. A comparison of DASS-21 results with psychiatric interviews showed that this tool had a sensitivity and specificity of 75% and 89%, respectively, and was capable of accurately screening depression, anxiety, and stress [28, 29].

- Maslach Burnout Inventory (MBI): The MBI is a 22-item questionnaire on a 5-points Likert scale that assesses the three theoretical components of burnout syndrome: emotional exhaustion "I feel emotionally drained from my work," depersonalization "I feel I treat some patients as if they were impersonal objects," and personal accomplishment "I deal very effectively with the problems of my patients" [25]. Higher scores in the emotional exhaustion and depersonalization scales indicate greater burnout, whereas higher scores in the personal accomplishment subscale indicate less burnout. Cutoffs for moderate and severe emotional exhaustion were ≥ 17 and ≥ 27, for moderate and severe depersonalization ≥ 7 and ≥ 13, and for moderate and severe reduced personal accomplishment ≤ 38 and ≤ 21 [30].

- CWEQ-II: is designed to measure four dimensions of empowerment – perceived access to opportunity, support, information and resources in an individual’s work setting – based on Kanter’s theory of structural empowerment (https://www.uwo.ca/fhs/hkl/cweq.html). The items were derived from Kanter's original ethnographic study of work empowerment and modified by Chandler (1986) for use in a nursing population. The CWEQ-II has been extensively studied and used in nursing research since 2000, demonstrating consistent reliability and validity. The overall empowerment score can range from 12 to 60, calculated by summing the scores of the first four subscales: (a) opportunity, (b) support, (c) information, and (d) resources. Scores ranging from 12 and 26 indicate low levels of an empowered work environment, 27 to 44 indicate moderate levels, and 46 to 60 indicate high levels of an empowered work environment [26].

### Statistical analysis

The normality of data distribution was evaluated using the Shapiro–Wilk test. Continuous variables with normal distribution were expressed as mean ± standard division (SD), while variables with non‑normal distributions were expressed as median (inter-quartile range, IQR). Categorical data were expressed as frequencies with percentage (%) and proportions. Sociodemographic characteristics and other COVID-9 related background data were compared between those working in the COVID-19 area (yes vs. no) using t‑test or Mann–Whitney test for normally and non-normally distributed variables, respectively. Chi-square tests or Fisher’s exact tests were used to compare the categorical data where appropriate. The total scores and subscales scores of DASS-21, MBI and CWEQ-II were expressed as median (IQR) and percentage for categorical groups based on cut-off points in all participants, as well as between groups who worked in the COVID-19 area or not. To compare median (IQR) scores of questionnaires, the Mann–Whitney test was applied. Unadjusted and adjusted binary logistic regression analysis were used to determine potential predictors for the total scores of DASS-21, MBI and CWEQ-II. Categories of total scores of DASS-21 and MBI questionnaires were expressed based on median. Multiple regression was used to adjust for confounders such as age, gender, having children, job position, working with COVID-19 patients, and history of mental health issues. For binary logistic regression, odds ratio (OR) and 95% confidence interval (CI) were calculated. GraphPad Prism 9© (GraphPad Software Inc., La Jolla, CA) was used to create a forest plot showing OR regression analysis. All analyses were conducted using SPSS software (ver.21) (SPSS Inc. IL, Chicago, USA) and a two-tailed *P*-value of < 0.05 was considered statistically significant.

## Results

### Responses rate

An online survey was sent via email to healthcare workers (HCWs) from 48 different countries. Out of the 1030 participants, all completed the sociodemographic section, resulting in a response rate of 100%. A total of 730 participants completed the DASS-21 questionnaire (70.9%), 851 completed the MBI questionnaire (82.6%), and 712 completed the CWEQ-II questionnaire (69.1%).

### Participants’ sociodemographic characteristics

The mean ± SD age of all responders (*n* = 1030) was 38.88 ± 9.63 years (range: 21–74 years) and 54.4% (*n* = 560) of them were male. The majority of participants were physicians (*n* = 562, 54.6%), followed by nurses (n = 279, 27.1%). Out of 1030 responders, 332 (32.2%) HCWs worked in ICU, 185 (18%) were from internal medicine, 118 (11.5%) were from emergency departments, and 109 (10.6%) were from anesthesiology. The majority of participants were working in Qatar (*n* = 400, 38.8%) and India (*n* = 161, 15.6%). The frequency of participants by other countries are available at Supplementary File 2 in Figure S1 and S2.

Among all responders (*n* = 1030), 763 (74.1%) of HCWs had been working in areas designated for COVID-19 patients. Out of the 763 HCWs, 692 (90.7%) had been directly involved in the care or management of COVID-19 patients for ≤ 9 months (*n* = 403/763, 52.8%) and for > 9 months (*n* = 360/763, 47.2%). During the survey period, 435 (42.2%) of HCWs received specific training for COVID-19, while 595 (57.85) did not. The sociodemographic characteristics of participants according to working in the COVID-19 area are presented in Table 1. The main significant differences between HCWs who worked in the COVID-19 area and those who did not were observed in terms of age (*P* < 0.001), specialty (*P* < 0.001), level of education (*P* = 0.008), working hours per week (*P* = 0.047), working hours per week during the COVID-19 pandemic (*P* < 0.001) and receipt of specific training (*P* = 0.034).

**Table 1:** Sociodemographic characteristics of participants according to working in COVID-19 area or not (*n* = 1030)

Sociodemographic characteristics		All participants (*n* = 1030)	Working in COVID-19 area		*P*-value
			**Yes (*n* = 763)**	**No (*n* = 267)**	
**Age (years)**	Mean ± SD	38.88 ± 9.63	38.30 ± 8.8	40.55 ± 11.38	0.001*
	Range	(21–74)	(22–66)	(21–74)	
**Gender**	Male (%)	560 (54.4)	420 (55.0)	140 (52.4)	0.461
	Female (%)	470 (45.6)	343 (45.0)	127 (47.6)	
**Marital status**	Single (%)	262 (25.4)	192 (25.2)	70 (26.2)	0.927
	Married (%)	731 (71.0)	543 (71.2)	188 (70.4)	
	Divorce/ Widowed (%)	37 (3.6)	28 (3.7)	9 (3.4)	
**Having children**	Yes (%)	654 (63.5)	478 (62.6)	176 (65.9)	0.339
	No (%)	376 (36.5)	285 (37.4)	91 (34.1)	
**Job position**	Nurse (%)	279 (27.1)	215 (28.2)	64 (24.0)	0.378
	Physician (%)	562 (54.6)	415 (54.4)	147 (55.1)	
	Therapist (%)	74 (7.2)	54 (7.1)	20 (7.5)	
	Others (%)	115 (11.2)	79 (10.4)	36 (13.5)	
**Specialty**	Anesthesiology (%)	109 (10.6)	78/ (10.2)	31 (11.6)	< 0.001*
	Internal medicine (%)	185 (18.0)	119 (15.6)	66 (24.7)	
	Critical care (%)	332/ (32.2)	294 (38.5)	38 (14.2)	
	Surgery (%)	25 (2.4)	16 (2.1)	9 (3.4)	
	Emergency (%)	118 (11.5)	102 (13.4)	16 (6.0)	
	Others (%)	261 (25.3)	154 (20.2)	107 (40.1)	
**Education levels**	Bachelors (%)	435 (42.2)	327 (42.9)	108 (40.4)	0.008*
	Masters (%)	227 (22.0)	173 (22.7)	54 (20.2)	
	PhDs (%)	78 (7.6)	45 (5.9)	33 (12.4)	
	Medical degree (MD) (%)	290 (28.2)	218 (28.6)	72 (27.0)	
**Work experience as HCW**	≤ 6 years (%)	568 (55.1)	419 (54.9)	149 (55.8)	0.801
	> 6 years (%)	462 (44.9)	344 (45.1)	118 (44.2)	
**Working hours per week**	≤ 27 h. (%)	517 (50.2)	369 (48.4)	148 (55.4)	0.047*
	> 27 h. (%)	513 (49.8)	394 (51.6)	119 (44.6)	
**Directly working with COVID-19 patients**	Yes (%)	692 (62.7)	692 (90.7)	0	< 0.001*
	No (%)	338 (32.8)	71 (9.3)	267 (100)	
**Work experience with COVID-19 patients**	No	267 (25.9)	0	267 (100)	< 0.001*
	≤ 9 months	403 (39.1)	403 (52.8)	0	
	> 9 months	360 (35.0)	360 (47.2)	0	
**Received specific training for COVID-19**	Yes (%)	435 (42.2)	337 (44.2)	98 (36.7)	0.034*
	No (%)	595 (57.8)	426 (55.8)	169 (63.3)	
**Working hours per week during the pandemic**	≤ 29 h. (%)	550 (53.4)	367 (48.1)	183 (68.5)	< 0.001*
	> 29 h. (%)	480 (46.6)	396 (51.9)	84 (31.5)	
**Taking care of COVID-19 patients in the last 24 h**	Yes (%)	368 (35.7)	368 (48.2)	0	< 0.001*
	No (%)	662 (64.3)	395 (51.8)	267 (100)	
**Last time caring for COVID-19 patients**	Never (%)	322 (31.2)	55 (7.2)	267 (100)	< 0.001*
	Last month (%)	627 (60.9)	627 (82.2)	0	
	≥ 3 months (%)	81 (7.9)	81 (10.6)	0	
**History of mental illness**	Yes (%)	87 (8.4)	69 (9.0)	18 (6.7)	0.244
	No (%)	943 (91.6)	694 (91.0)	249 (93.3)	
**Current use of medication for mental illness**	Yes (%)	56 (5.4)	39 (5.1)	17 (6.4)	0.436
	No (%)	974 (94.6)	724 (94.9)	250 (93.6)	
**Family history of mental illness**	Yes (%)	118 (11.5)	83 (10.9)	35 (13.1)	0325
	No (%)	912 (88.5)	680 (89.1)	232 (86.9)	

^*^P < 0.05 considered as significant

### Total and subscales scores of questionnaires

**Fig. 1:**
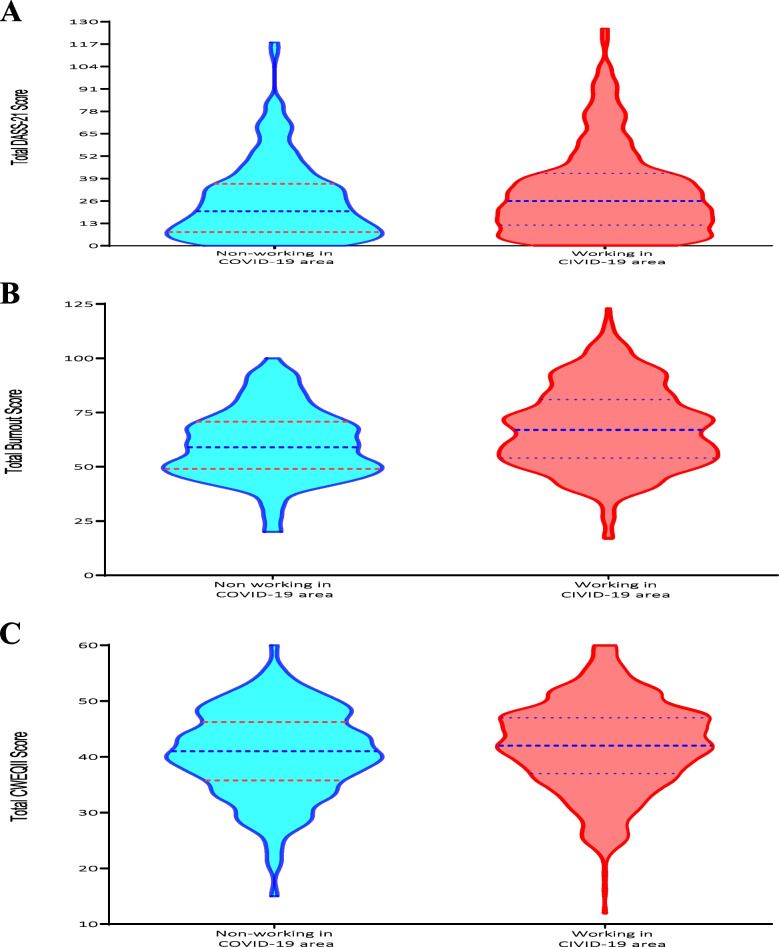
Total scores of (**A**) DASS-21, (**B**) MBI and (**C**) CWEQ-II according to HCWs who worked in COVID-19 area or not were expressed as median (IQR)

Total and subscale scores of the DASS-21, MBI and CWEQ-II scales in all participants, as well as in HCWs who worked in the COVID-19 area or not, are presented in Table 2. Among all responders (*n* = 730), the median (IQR) scores of stress, anxiety and depression were 12 (6–18), 6 (2–12), and 6 (2–14), respectively. The results of subscale scores based on categories groups showed that the majority of HCWs had normal level of stress (*n* = 364, 49.9%), anxiety (*n* = 391, 53.6%) and depression (*n* = 433, 59.3%). The median (IQR) scores of emotional exhaustion, depersonalization and personal accomplishment in all responders (*n* = 852) were 22 (11–32), 6 (3–11) and 37 (31–42), respectively. The results of categorized subscales indicated that the HCWs experienced high emotional exhaustion, while low depersonalization and personal accomplishment according to MBI scale. Furthermore, the four elements of CWEQ-II showed that HWCs believed they had moderate access to opportunity and information, with median (IQR) scores of 12 (10–14) and 11 (9-12), respectively, and a low access to support and resources, with a score of 10 (9–12) and 9 (8–11), respectively. In addition, the median (IQR) total scores of DASS-21, MBI and CWEQ-II according to the HCWs who worked in the COVID-19 area or did not work in the COVID-19 area are presented in Fig. 1A to C. According to these figures, the median (IQR) of total scores of DASS-21, MBI and CWEQ-II were significantly higher in the HCWs who worked in COVID-19 area.

**Table 2:** The scores of questionnaires from HCWs according working in COVID-19 area or not (*n* = 730)

Score of questionnaires		All participants	Working in COVID-19 area		*P*-value
			**Yes**	**No**	
**Depression Anxiety and Stress Scale 21 (DASS-21)**		***n* = 730**	***n* = 555**	***n* = 175**	
**DASS-21 Stress**	Median (IQR)	12 (6–18)	12 (6–18)	10 (4–14)	0.078
	Normal (0–10) (%)	364 (49.9)	271 (48.8)	93 (53.1)	
	Mild (11–18) (%)	226 (31)	175 (31.5)	51 (29.1)	
	Moderate (19–26) (%)	77 (10.5)	58 (10.5)	19 (10.9)	
	Severe (27–34) (%)	48 (6.6)	36 (6.5)	12 (6.9)	
	Extremely severe (35–42) (%)	15 (2.1)	15 (2.7)	0	
**DASS-21 Anxiety**	Median (IQR)	6 (2–12)	6 (2–12)	4 (2–10)	0.005*
	Normal (0–6) (%)	391 (53.6)	282 (50.8)	109 (62.3)	
	Mild (7–9) (%)	76 (10.4)	61 (11)	15 (8.6)	
	Moderate (10–14) (%)	142 (19.5)	111 (20)	31 (17.7)	
	Severe (15–19) (%)	42 (5.8)	35 (6.3)	7 (4)	
	Extremely severe (20–42) (%)	79 (10.8)	66 (11.9)	13 (7.4)	
**DASS-21 Depression**	Median (IQR)	6 (2–14)	6 (2–14)	6 (0–12)	0.040*
	Normal (0–9) (%)	433 (59.3)	323 (58.2)	110 (62.9)	
	Mild (10–12) (%)	108 (14.8)	80 (14.4)	28 (16)	
	Moderate (13–20) (%)	103 (14.1)	79 (14.2)	24 (13.7)	
	Severe (21–27) (%)	39 (5.3)	31 (5.6)	8 (4.6)	
	Extremely severe (28–42) (%)	47 (6.4)	42 (7.6)	5 (2.9)	
**Total score of DASS-21**	Mean ± SD	24 (12–40)	26 (12–42)	20 (8–36)	0.016*
	Normal-Moderate (≤ 24) (%)	370 (50.7)	271 (48.8)	99 (56.6)	
	Severe/extremely severe (> 24) (%)	360 (49.3)	284 (51.2)	76 (43.4)	
**Maslach Burnout Inventory (MBI) questionnaire**		***n* = 852**	***n* = 640**	***n* = 212**	
**Emotional exhaustion**	Median (IQR)	22 (11–32)	23 (12–34)	18 (8–27)	< 0.001*
	Low (0–16) (%)	328 (38.5)	232 (36.3)	96 (45.3)	
	Moderate (17–26) (%)	194 (22.8)	132 (20.7)	62 (29.2)	
	High (≥ 27) (%)	329 (38.7)	275 (43)	54 (25.5)	
**Depersonalization**	Median (IQR)	6 (3–11)	6 (3–12)	5 (2–9)	< 0.001*
	Low (0–6) %)	452 (53.1)	322 (50.4)	130 (61.3)	
	Moderate (7–12) (%)	226 (26.6)	169 (26.4)	57 (26.9)	
	High (≥ 13) (%)	173 (20.3)	148 (23.2)	25 (11.8)	
**Personal accomplishment**	Median (IQR)	37 (31–42)	37 (31–42)	37 (31–42)	0.961
	Low (≥ 39) (%)	356 (41.8)	262 (41)	94 (44.3)	
	Moderate (32–38) (%)	280 (32.9)	216 (33.8)	64 (30.2)	
	High (0–31) (%)	215 (25.3)	161 (25.2)	54 (25.5)	
**Total score of MBI**	Median (IQR)	64 (52–78)	67 (54–81)	59 (49–71)	< 0.001*
	Low-Moderate (≤ 64) (%)	429 (50.4)	297 (46.5)	132 (62.3)	
	High (> 64) (%)	422 (49.6)	342 (53.5)	80 (37.7)	
**Conditions for work effectiveness questionnaire (CWEQ-II)**		***n* = 712**	***n* = 538**	***n* = 174**	
**Opportunity**	Median (IQR)	12 (10–14)	12 (10–14)	11 (9–13)	< 0.001*
	Low-Moderate (≤ 12) (%)	399 (56.0)	280 (52.0)	119 (68.4)	
	High (> 12) (%)	313 (44.0)	258 (48.0)	55 (31.6)	
**Information**	Median (IQR)	11 (9–12)	11 (9–12.25)	11 (9–12.25)	0.527
	Low-Moderate (≤ 11) (%)	416 (58.4)	324 (60.2)	92 (52.9)	
	High (> 11) (%)	296 (41.6)	214 (39.8)	82 (47.1)	
**Support**	Median (IQR)	10 (9–12)	10 (9–12)	10 (9–12)	0.635
	Low-Moderate (≤ 10) (%)	434 (61.0)	322 (59.9)	112 (64.4)	
	High (> 10) (%)	278 (39.0)	216 (40.1)	62 (35.6)	
**Resources**	Median (IQR)	9 (8–11)	10 (8–11)	9 (9–11)	0.726
	Low-Moderate (≤ 9) (%)	362 (50.8)	268 (49.8)	94 (54.0)	
	High (> 9) (%)	350 (49.2)	270 (50.2)	80 (46.0)	
**Total score of CWEQ-II **	Median (IQR)	42 (37–47)	42 (37–47)	41 (37.75–46.25)	0.055
	Low-Moderate (12–44) (%)	444 (62.4)	321 (59.7)	123 (70.7)	0.009*
	High (45–60) (%)	268 (37.6)	217 (40.3)	51 (29.3)	

^*^P < 0.05 considered as significant, Categories of total score of DASS-21, MBI and subscales of CWEQ-II questionnaire were expressed based on median (IQR)

The median (IQR) scores of the DASS-21, MBI and CWEQ-II scales were compared between the groups of HCWs who worked in the COVID-19 area or not. The results showed that the median score of anxiety (*P* = 0.005), depression (*P* = 0.040) and total score of DASS-21 (*P* = 0.016), in HCWs who worked in the COVID-19 area were significantly higher than those who did not work in the COVID-19 area. Moreover, HCWs who worked in the COVID-19 area had a significantly higher median emotional exhaustion (*P* < 0.001), depersonalization (*P* < 0.001) and total score of MBI (*P* < 0.001) compared to those who did not work in the COVID-19 area. In terms of CWEQ-II, HCWs who worked in COVID-19 areas had a significant higher score in opportunity (*P* < 0.001).

### Regression analysis findings

**Fig. 2:**
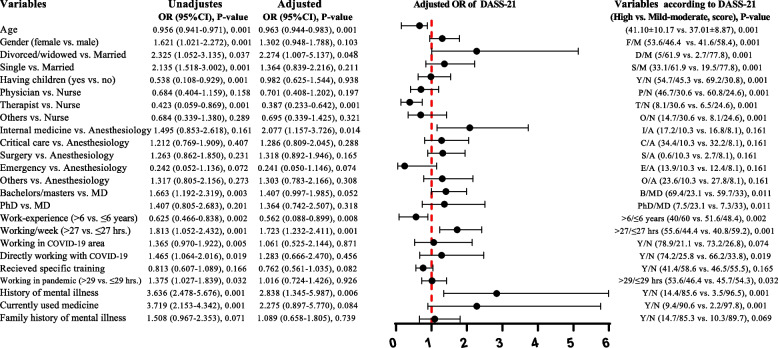
Unadjusted and adjusted binary logistic regression analysis of DASS-21 prognostic total scores. Forest plot showed results, after adjusting for the factors: age, gender, having children, job position, working in COVID-19 area and history of mental health issues. In addition, a comparison of respondents' demographic variables based on high versus low-moderate DASS-21 scores is reported. Abbreviations; F/M: female/male; D/M: divorced/widowed/married; S/M: single/married, Y/N: yes/no; P/N: physician/nurse; T/N: therapist/nurse; O/N: others/nurse; I/A: internal medicine/anesthesiology; C/A: critical care/anesthesiology; S/A: surgery/anesthesiology; E/A emergency/anesthesiology; O/A others/anesthesiology; B/MD: bachelors-masters/ doctor of medicine; PhD/MD: doctor of philosophy/ doctor of medicine and OR: odds ratio

**Fig. 3:**
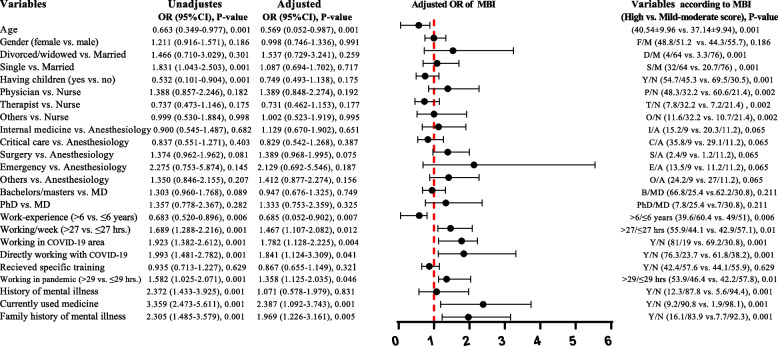
Unadjusted and adjusted binary logistic regression analysis of MBI prognostic total scores. Forest plot showed results, after adjusting for the factors: age, gender, having children, job position, working in COVID-19 area and history of mental health issues. In addition, a comparison of respondents' demographic variables based on high versus low-moderate MBI scores is reported. Abbreviations; F/M: female/male; D/M: divorced/widowed/married; S/M: single/married, Y/N: yes/no; P/N: physician/nurse; T/N: therapist/nurse; O/N: others/nurse; I/A: internal medicine/anesthesiology; C/A: critical care/anesthesiology; S/A: surgery/anesthesiology; E/A emergency/anesthesiology; O/A others/anesthesiology; B/MD: bachelors-masters/ doctor of medicine; PhD/MD: doctor of philosophy/ doctor of medicine and OR: odds ratio

**Fig. 4:**
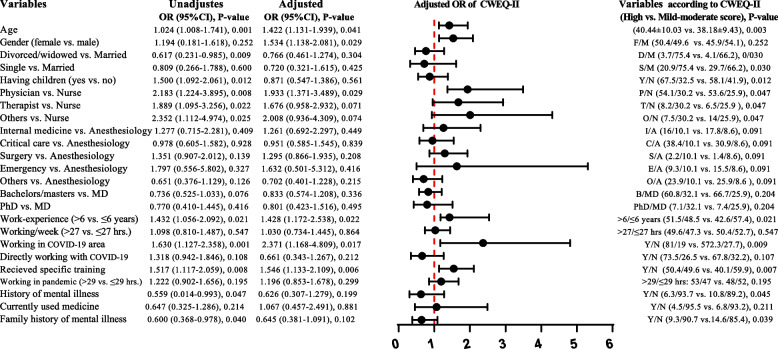
Unadjusted and adjusted binary logistic regression analysis of CWEQ-II prognostic total scores. Forest plot showed results, after adjusting for the factors: age, gender, having children, job position, working in COVID-19 area and history of mental health issues. In addition, a comparison of respondents' demographic variables based on high versus low-moderate CWEQ-II scores is reported. Abbreviations; F/M: female/male; D/M: divorced/widowed/married; S/M: single/married, Y/N: yes/no; P/N: physician/nurse; T/N: therapist/nurse; O/N: others/nurse; I/A: internal medicine/anesthesiology; C/A: critical care/anesthesiology; S/A: surgery/anesthesiology; E/A emergency/anesthesiology; O/A others/anesthesiology; B/MD: bachelors-masters/ doctor of medicine; PhD/MD: doctor of philosophy/ doctor of medicine and OR: odds ratio

Unadjusted and adjusted binary logistic regression analysis were conducted to determine potential predictors for the total scores of DASS-21, MBI and CWEQ-II. The results are presented in Figs. 2, 3, and 4.

Adjusted binary logistic regression analysis for the prognostic value DASS-21 (Fig. 2) showed that the divorced/ widowed HCWs (OR: 2.274, 95% CI: 1.007–5.137, *P* = 0.048), those working in internal medicine (OR: 2.077, 95% CI: 1.157–3.726, *P* = 0.014), those working more than 27 h per week (OR: 1.723, 95% CI: 1.232–2.411, *P* = 0.001) and those with a history of mental illness (OR: 2.838, 95% CI: 1.345–5.987, *P* = 0.006) had a higher likelihood of experiencing stress, anxiety and depression in comparison to married HCWs, specifically those in anesthesiology, working ≤ 27 h per week, and those without history of mental illness, respectively. However, higher age (OR: 0.663, 95% CI: 0.144–0.883, *P* = 0.001) and higher work experience of more than 6 years (OR: 0.562, 95% CI: 0.088–0.899, *P* = 0.008) were found to be negatively associated with the total score of DASS-21.

Adjusted binary logistic regression analysis for the prognostic value MBI (Fig. 3) revealed that older HCWs (OR: 0.569, 95% CI: 0.052–0.887, *P* = 0.001) and those with higher work experience of more than 6 years (OR: 0.585, 95% CI: 0.052–0.802, *P* = 0.007) had a lower likelihood of experiencing burnout compared to younger HCWs and those with less work experience. While, working longer than 27 h per week (OR: 1.467, 95% CI: 1.107–2.082, *P* = 0.012), working more than 29 h per week during the COVID-19 outbreak (OR: 1.358, 95% CI: 1.125–2.035, *P* = 0.046), working in COVID-19 area within the hospital (OR:1.782, 95% CI: 1.128–2.225, *P* = 0.004), directly interacting with COVID-19 patients (OR: 1.841, 95% CI: 1.124–3.309, *P* = 0.041), currently taking medication for mental illness (OR: 2.387, 95% CI: 1.192–3.743, *P* = 0.001) and having a family history of mental illness (OR: 1.969, 95% CI: 1.226–3.161, *P* = 0.005) were positively associated with burnout among HCWs.

Adjusted binary logistic regression was applied to the prognostic CWEQ-II (Fig. 4), indicating that age (OR: 1.422, 95% CI: 1.131–1.039, *P* = 0.041), female gender (OR: 1.534, 95% CI: 1.138–2.081, *P* = 0.029), physicians (OR: 1.933, 95% CI: 1.371–3.489, *P* = 0.029), higher work experience (OR: 1.428, 95% CI: 1.172–2.538, *P* = 0.022), working in the COVID-19 area (OR: 2.371, 95% CI: 1.168–4.809, *P* = 0.017) and receiving specific training (OR: 1.546, 95% CI: 1.133–2.109, *P* = 0.006) were positively correlated with work effectiveness.

## Discussion

The main aims of this cross-sectional web-based questionnaire survey were to assess the psychological distress (stress, anxiety and depression), burnout and structural empowerment status among HCWs during the COVID- 19 pandemic and to evaluate their predictors. The results showed that 360 out of 712 (49.3%) responders experienced severe or extremely severe levels of stress, anxiety, and depression; 422 out of 851 (49.6%) responders reported a high level of burnout, and 268 out of 712 (37.6%) responders indicated a high level of structural empowerment based on the DASS-21, MBI, and CWEQ-II scales, respectively. Regarding working in an empowered work environment, more than half of the responders reported low-to-moderate access to opportunity (*n* = 399, 56%), information (*n* = 416, 58.4%), support (*n* = 434, 61%), and resources (*n* = 362, 50.8%). Further analysis of the DASS-21, BMI and CWEQ-II scores was conducted by dividing the participants into two groups: those working in areas designated for COVID-19 patients (74.1%), and those not working in such areas (25.9%). The analysis showed that HCWs caring for COVID-19 patients experienced significantly higher symptoms of severe stress, anxiety, depression and higher levels of burnout compared to those working in other areas. The results also revealed that direct work with COVID-19 patients, lower work experience, and high workload during the outbreak of COVID-19 increased the risks of negative psychological consequences.

Compared to other published data, our findings showed a similar prevalence of depression, anxiety, and stress among HCWs from Qatar, with rates of 12.4%, 14.2%, and 18.5%, respectively [31]. In another study conducted on medical residents in Qatar, the results showed higher prevalence rates of depression, anxiety, and stress, with 42.5%, 41.7% and 30.7% of all participants experiencing these conditions, respectively [32]. The results of the present study along with previous studies demonstrate the massive impact of the pandemic on the psychological health of healthcare professionals. COVID-19 has imposed irreversible psychological impacts on HCWs due to rapid changes in medical information and procedures, their self-perception of risk, the pandemic's influence on their lifestyle, long working hours, and separation from their families [33]. One of the demographic factors predicting mental distress in our study were younger age and being single or divorced/widowed as compared to married HCWs. This finding is consistent with previous studies, which have shown that although older people are more susceptible to COVID-19 [34], younger individuals are more prone to psychological distress. This may be attributed to weaker resilience factors and fewer resources in the face of a crisis [35]. Loneliness and isolation are additional factors that make HCWs prone to stress and anxiety. Our findings, in line with previous studies, indicated that a lack of social support is related to mental health problems [36, 37]. Family members typically serve as the main source of support and not having a spouse or life partner during similar situations augments the level of stress [38].

Results of the current survey showed that the majority of participants reported moderate to severe levels of emotional exhaustion (61.5%) and reduced personal accomplishment (74.7%). Additionally, nearly half of the participants (46.9%) reported moderate to severe levels of depersonalization. High levels of burnout among physicians were supported by Hu et al. [39], Jalili et al. [40], and Orrù et al. [41], who also assessed MBI-based burnout. In line with previous studies, we found that the main reasons for burnout in HCWs were working directly with COVID-19 patients and high workload [42, 43]. These reasons could be attributed to a greater fear of infection, lack of sufficient time to recover and inadequate hospital facilities. Burnout is associated with increased risks of both physical and psychological long-term detrimental consequences [44]. Furthermore, it is linked to increased sick leave, absenteeism, job withdrawal, and poor work efficiency [44]. Given the potential extended duration of the pandemic [45], the negative impact of the high prevalence of burnout may exacerbate and reduce the capacity of health systems to cope with the increased demand of care likely to occur in both the short- and long-term [42].

To evaluate the status of structural empowerment, the scores of four elements of CWEQ-II were compared among HCWs in this survey. Overall, HCWs from COVID-19 areas scored higher in most elements; particularly in the areas of opportunity, support and resources. Notably, HCWs working in COVID-19 designated areas reported significantly greater access to opportunities compared to those working in other areas. The results showed that dealing with COVID-19 patients provided chances for HCWs to grow and enhance their knowledge and skills. The opportunity to care for COVID-19 patients yielded a range of emotions for HCWs, exposed advancements and gaps in their preparation, and challenged them to independently develop new care practices and processes. This development of innovative practices and processes allowed them to explore new ideas in patient care and take proactive measures in treating COVID-19 patients. They used this opportunity to make an impact on patient care and also provided recommendations for changes [46].

Although our study provides useful insight into the mental health status of HCWs, we recognize a few limitations. Firstly, the data was obtained using a self-reported questionnaire and was not validated by medical records. Secondly, its cross-sectional nature and early assessment limit the ability to determine long-term effects of the pandemic. Finally, the sample size for countries other than Qatar is not sufficient, which may limit the generalizability of the results. Follow-up studies would be beneficial to assess the psychological manifestations of the pandemic, considering the current improvement in knowledge and strategy to deal with COVID-19. Notwithstanding the aforementioned limitations, our study contributes to the literature by providing information about the psychological effects of COVID-19 in HCWs from different job positions, specialties, and regions. Furthermore, our study identifies vulnerable groups who are more susceptible to psychological distress. Thus, development of a psychological support system targeting these groups would be useful to maintain the wellbeing of the HCWs.

In conclusion, this study shows that health professionals have a high risk of incurring in burnout or psychological conditions due to the COVID-19 pandemic. Continuous monitoring and timely treatment of these conditions is needed to preserve the professionals’ health and to enhance the healthcare systems preparedness to face the medium- and long-term consequences of the outbreak.

### Supplementary Information

**Additional file 1.** Demographic questionnaire

**Additional file 2: Figure 1s.** Frequency (%) of healthcare workers enrollment in this survey from different countries, except participants from Qatar (*n*=400, 38.8%) and India (*n*=161, 15.6%). **Figure 2s.** Frequency (%) of healthcare workers enrollment in this survey from different countries, except participants from Qatar (*n*=400, 38.8%) and India (*n*=161, 15.6%), according to HCWs who worked in COVID-19 area or not.

## Data Availability

The data that support the findings of this study are available from the corresponding author upon reasonable request.

## Abbreviations

COVID-19: Coronavirus disease 2019
HCWs: Healthcare workers
DASS-21: Depression Anxiety and Stress Scale 21
SE: Structural empowerment
MBI: Maslach Burnout Inventory
CWEQ_II: Conditions for work effectiveness questionnaire
SARS-CoV-2: Severe acute respiratory syndrome coronavirus 2
PTSD: Posttraumatic stress disorder
PPE: Personal protective equipment
ICU: Intensive care unit
STROBE: Strengthening the Reporting of Observational Studies in Epidemiology
