# Supplementary material for: Evaluation of psychological distress, burnout and structural empowerment status of healthcare workers during the outbreak of coronavirus disease (COVID-19): a cross-sectional questionnaire-based study
Source: BMC Psychiatry. 2024 Jan 22;24:61. doi: 10.1186/s12888-023-05088-x (PMC10804486; doi:10.1186/s12888-023-05088-x)
Supplement: Supplementary file 1 — Additional file 1. Demographic questionnaire [file 12888_2023_5088_MOESM1_ESM.doc]

**Demographic questionnaire:**

- Gender: □ Male □ Female

- Age: ___ years

- Status: □ Single □ Married □ Divorced/ Widowed
- Children: □ Yes □ No

- What is your position: □ Nurse □ Paramedic □ Emergency physician

□ Resident/ trainee doctor □ Intensivist □ Anesthetist
□ Respiratory therapist □ Surgeon □ Physiotherapist □ Dietitian

□ Others ____________

- What is your place work: □ ED □ ICU □ ID □ Surgery

□ Trauma center □ Burn unit □ Nursing department
□ Laboratory □ Radiology □ Cardiology □ Pulmonary □ ENT □ Neurology □ Oncology □ Maternity □ Endocrinology
□ Orthopedics □ Dermatology □ Psychiatry □ Rehabilitation
□ Dentistry □ Physical therapy □ Other medical wards _____________

- What is your highest level of education? □ Bachelors □ Masters □ Doctorate
- For how long have you been working in this hospital? ______

- How many hours do your work per week? ______

- Have you been working in an area designated for COVID-19 patients?

□ Yes □ No

- Have you been directly involved in the care or management of COVID-19 patients?

□ Yes □ No

- If yes:
  - For how long have you been directly involved in the management of COVID-19 patients? ____ month _____ weeks
  - Did you undergo specific training for COVID-19? □ Yes □ No

- Have you been taking care of a COVID-19 patient over the last 24 hours?

□ Yes □ No

- If No, when was the last time?

□ Last week □ Last two weeks □ Last three weeks
 □ Last month □ Last two months □ Last three months
 □ More than four months ago

**MBI-HSS:**

The purpose of the following survey is to discover how various persons in the medical professions view their job and the people with whom they work closely.

*Instructions:* On the following pages are 22 statements of job-related feelings. Please read each statement carefully and decide if you ever feel this way about your job. If you have never had this feeling, select the Never option. If you have had this feeling, indicate how often you feel it by selecting the option that best describes how frequently you feel that way.

The phrases describing the frequency are:

Never A few times a year or less Once a month or less A few times a month

Once a week A few times a week Every day

1. I feel emotionally drained from my work.


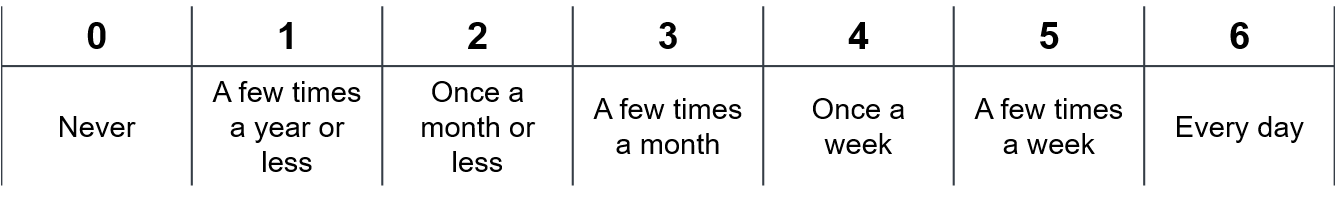


2. I feel used up at the end of the workday.


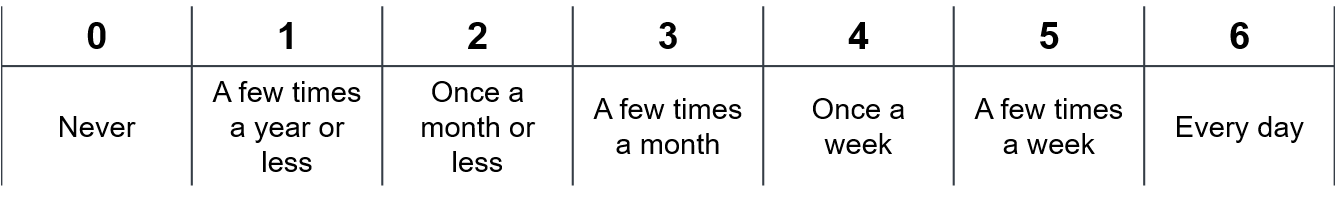


3. I feel fatigued when I get up in the morning and have to face another day on the job.


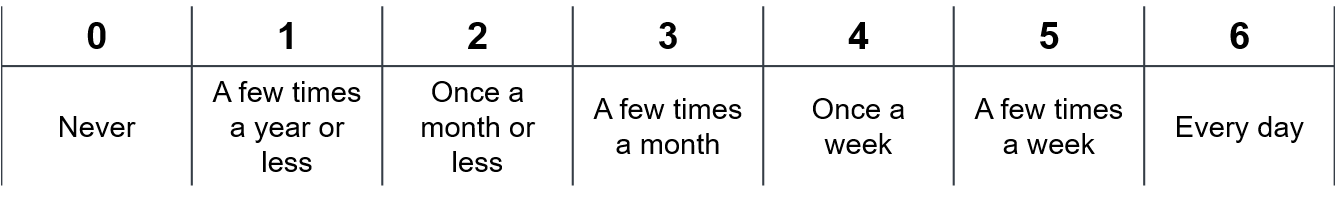


4. I can easily understand how my patients feel about things.


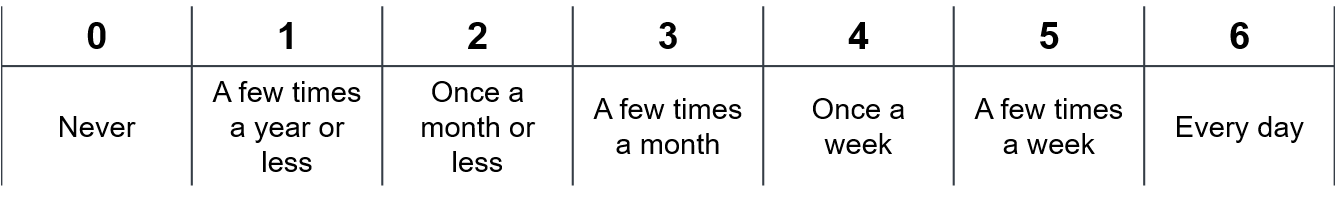


5. I feel I treat some patients as if they were impersonal objects.


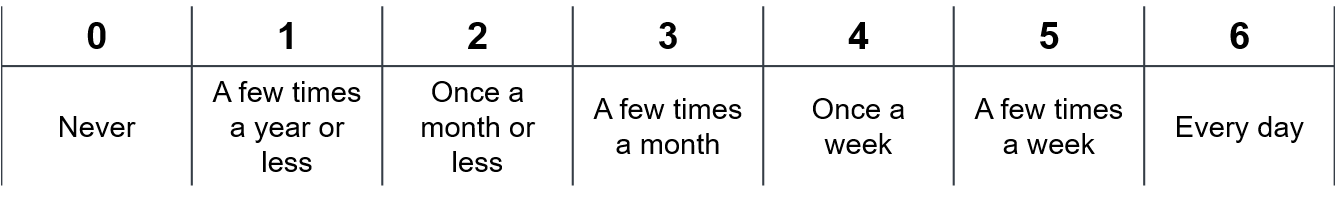


6. Working with people all day is really a strain for me.


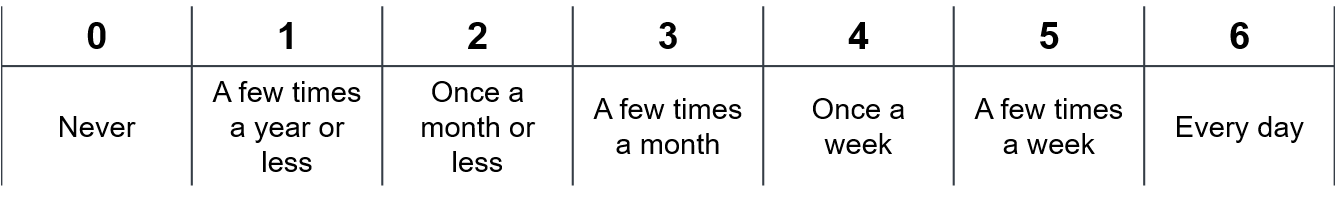


7. I deal very effectively with the problems of my patients.


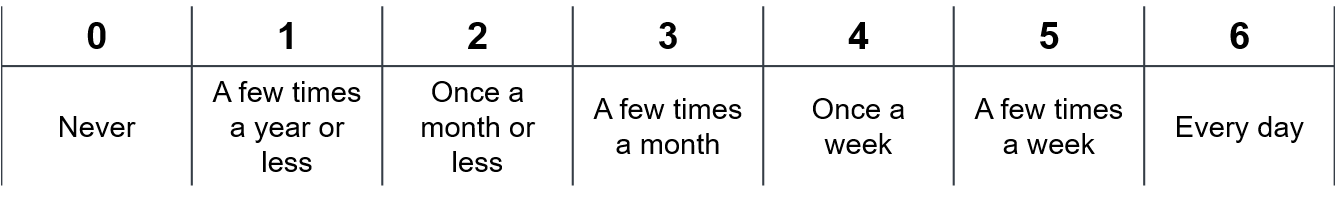


8. I feel burned out from my work.


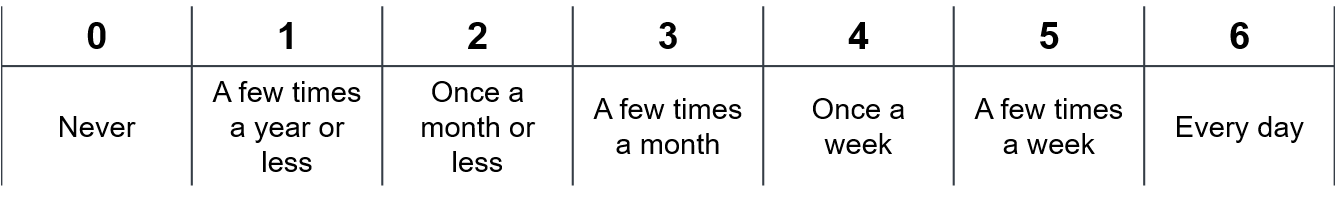


9. I feel I'm positively influencing other people's lives through my work.


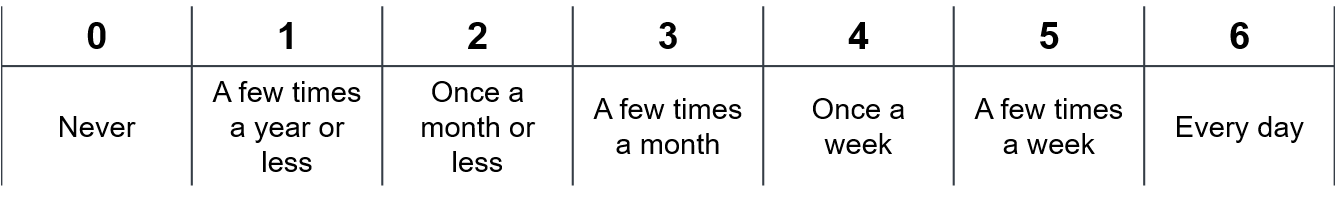


10. I've become more callous toward people since I took this job.


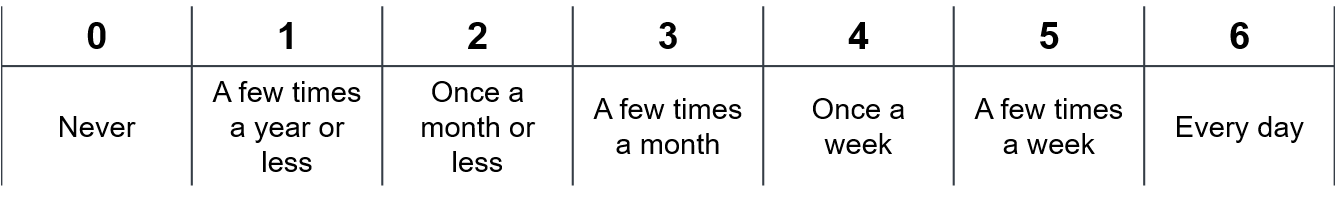


11. I worry that this job is hardening me emotionally.


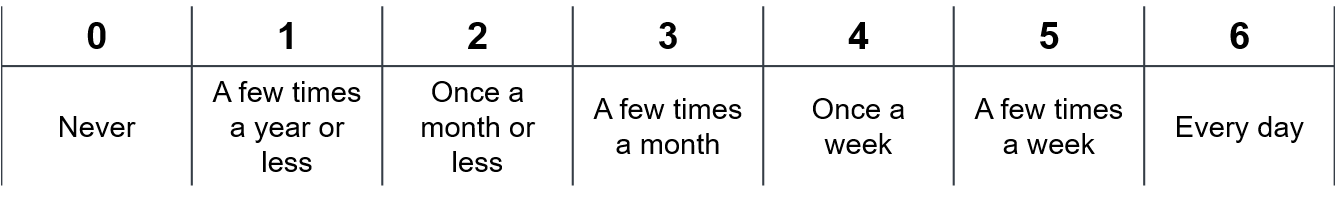


12. I feel very energetic.


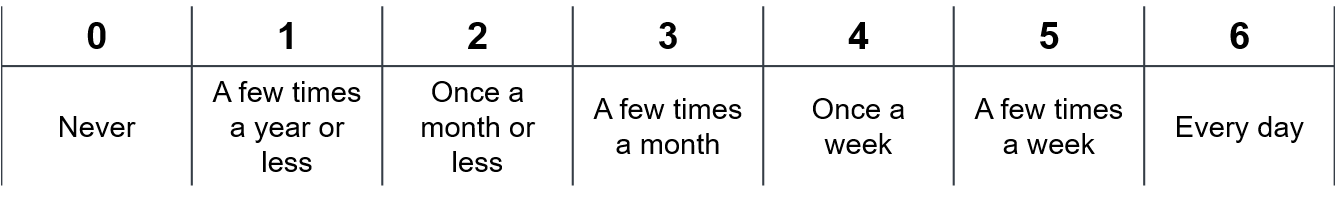


13. I feel frustrated by my job.


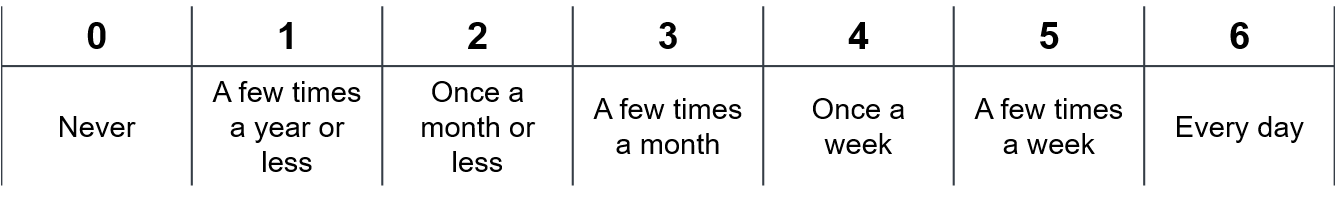


14. I feel I'm working too hard on my job.


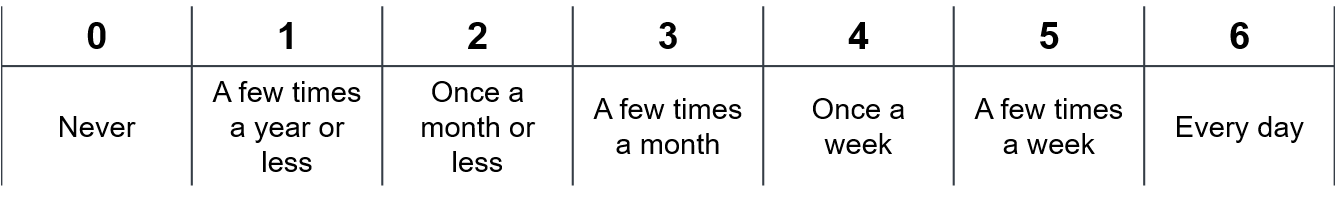


15. I don't really care what happens to some patients.


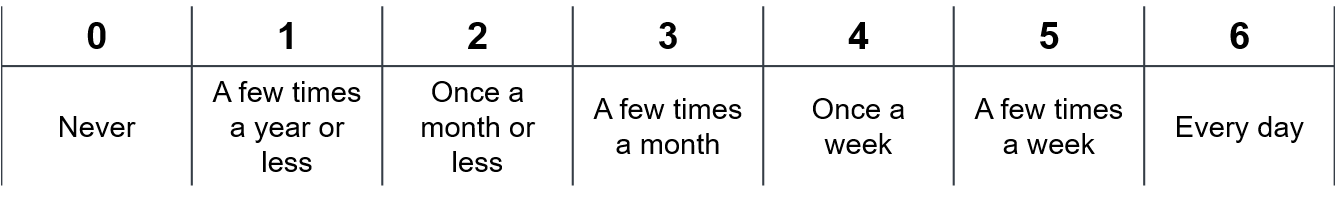


16. Working with people directly puts too much stress on me.


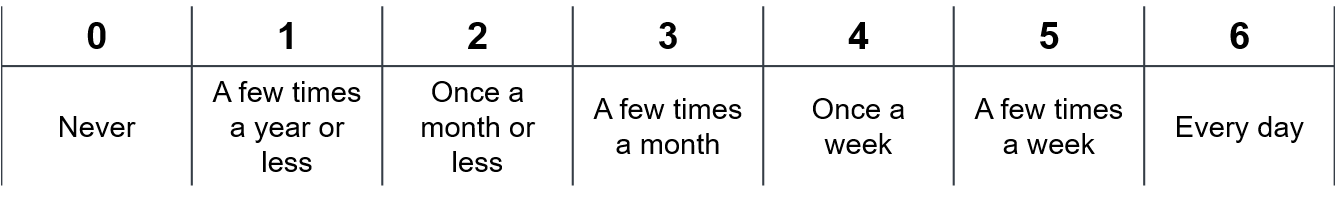


17. I can easily create a relaxed atmosphere with my patients.


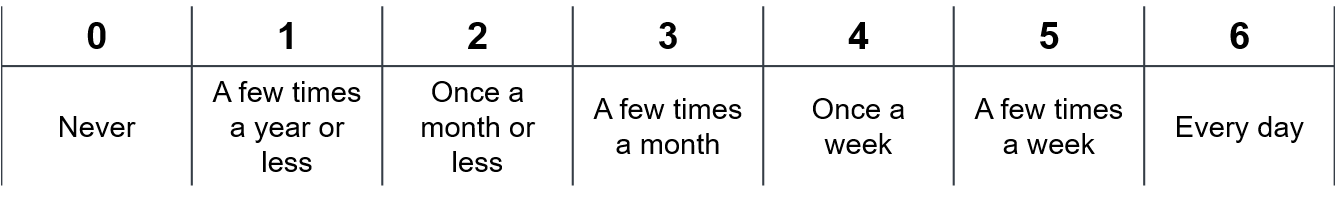


18. I feel exhilarated after working closely with my patients.


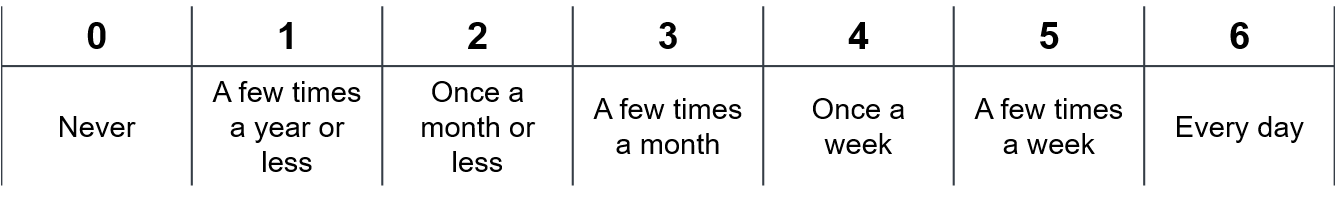


19. I have accomplished many worthwhile things in this job.


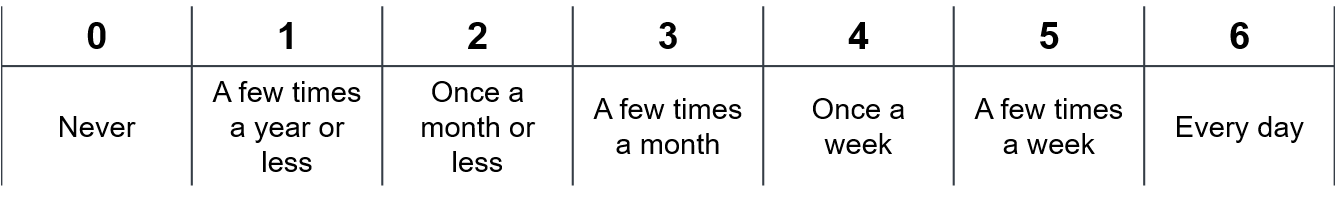


20. I feel like I'm at the end of my rope.


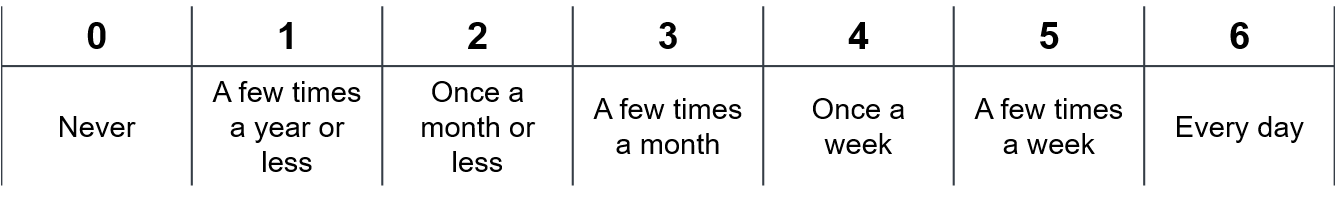


21. In my work, I deal with emotional problems very calmly.


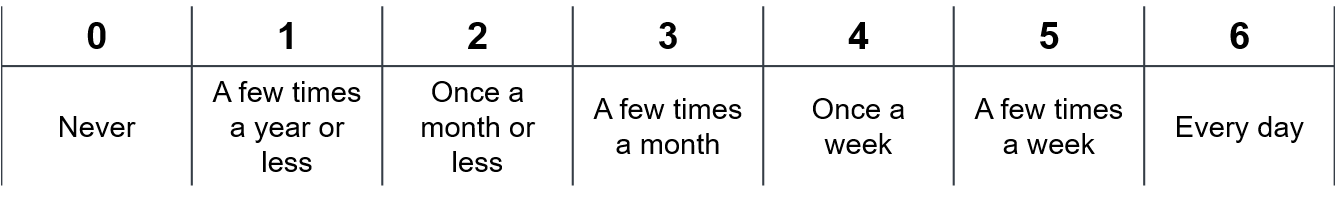


22. I feel patients blame me for some of their problems.


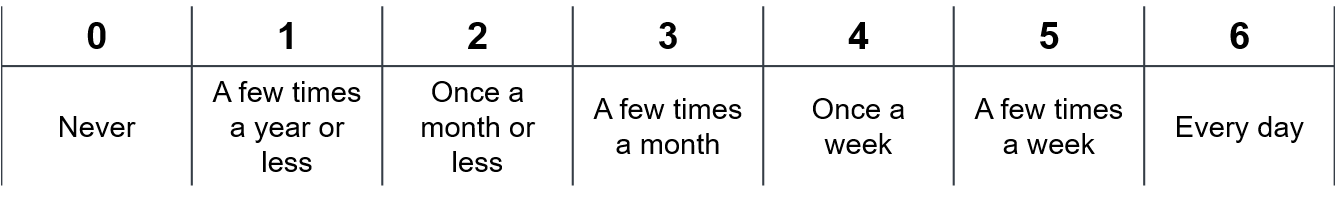


Copyright © 2012, 2016, 2018 by Christina Maslash and Sudan E. Jackson. All rights preserved. Published by Mind Garden, Inc. [www.mindgarden.com](http://www.mindgarden.com/) [v3.4]

**Depression Anxiety and Stress Scale (DASS-21):**

Please read each statement and select a number 0, 1, 2, 3 which indicates how much the statement applied to you **over the past week**. There are no right or wrong answers. Do not spend too much time on any statement.

**The rating scale is as follows:**

0 Did not apply to me at all

1 Applied to me to some degree, or some of the time

2 Applied to me to a considerable degree or a good part of time

3 Applied to me very much or most of the time

1 (s) I found it hard to wind down

0 1 2 3

2 (a) I was aware of dryness of my mouth

0 1 2 3

3 (d) I couldn’t seem to experience any positive feeling at all

0 1 2 3

4 (a) I experienced breathing difficulty (e.g. excessively rapid breathing breathlessness in the absence of physical exertion)

0 1 2 3

5 (d) I found it difficult to work up the initiative to do things

0 1 2 3

6 (s) I tended to over-react to situations

0 1 2 3

7 (a) I experienced trembling (e.g. in the hands)

0 1 2 3

8 (s) I felt that I was using a lot of nervous energy

0 1 2 3

9 (a) I was worried about situations in which I might panic and make a fool of myself

0 1 2 3

10 (d) I felt that I had nothing to look forward to

0 1 2 3

11 (s) I found myself getting agitated

0 1 2 3

12 (s) I found it difficult to relax

0 1 2 3

13 (d) I felt down-hearted and blue

0 1 2 3

14 (s) I was intolerant of anything that kept me from getting on with what I was doing

0 1 2 3

15 (a) I felt I was close to panic

0 1 2 3

16 (d) I was unable to become enthusiastic about anything

0 1 2 3

17 (d) I felt I wasn’t worth much as a person

0 1 2 3

18 (s) I felt that I was rather touchy

0 1 2 3

19 (a) I was aware of the action of my heart in the absence of physical exertion (e.g. sense of heart rate increase heart missing a beat)

0 1 2 3

20 (a) I felt scared without any good reason

0 1 2 3

21 (d) I felt that life was meaningless

0 1 2 3

Lovibond, S.H. & Lovibond, P.F. (1995). Manual for the Depression Anxiety & Stress Scales. (2nd Ed.) Sydney: Psychology Foundation.

**Conditions for Work Effectiveness Questionnaire-II (CWEQ-II):**

**How much of each kind of opportunity do you have in your present job?**

1 = None 2 3 = Some 4 5 = A Lot

1. Challenging work

1 2 3 4 5

1. The chance to gain new skills and knowledge on the job

1 2 3 4 5

1. Tasks that use all of your own skills and knowledge

1 2 3 4 5

**How much access to information do you have in your present job?**

1 = No Knowledge 2 3 = Some Knowledge 4 5 = Know A Lot

1. The current state of the hospital

1 2 3 4 5

1. The values of top management

1 2 3 4 5

1. The goals of top management

1 2 3 4 5

**How much access to support do you have in your present job?**

1 = None 2 3 = Some 4 5 = A Lot

1. Specific information about things you do well

1 2 3 4 5

1. Specific comments about things you could improve

1 2 3 4 5

1. Helpful hints or problem-solving advice

1 2 3 4 5

**How much access to resources do you have in your present job?**

1 = None 2 3 = Some 4 5 = A Lot

1. Time available to do necessary paperwork

1 2 3 4 5

1. Time available to accomplish job requirements

1 2 3 4 5

1. Acquiring temporary help when needed

1 2 3 4 5

Laschinger, H.K.S., Finegan, J., Shamian, J., &amp; Wilk, P. (2001). Impact of structural and

psychological empowerment on job strain in nursing work settings: Expanding Kanter’s model.

Journal of Nursing Administration, 31(5), 260-272.
