# Supplementary material for: Evaluation of psychological distress, burnout and structural empowerment status of healthcare workers during the outbreak of coronavirus disease (COVID-19): a cross-sectional questionnaire-based study
Source: BMC Psychiatry. 2024 Jan 22;24:61. doi: 10.1186/s12888-023-05088-x (PMC10804486; doi:10.1186/s12888-023-05088-x)
Supplement: Supplementary file 2 — Additional file 2: Figure 1s. Frequency (%) of healthcare workers enrollment in this survey from different countries, except participants from Qatar (n=400, 38.8%) and India (n=161, 15.6%). Figure 2s. Frequency (%) of healthcare workers enrollment in this survey from different countries, except participants from Qatar (n=400, 38.8%) and India (n=161, 15.6%), according to HCWs who worked in COVID-19 area or not. [file 12888_2023_5088_MOESM2_ESM.docx]

**Figure 1s:** Frequency (%) of healthcare workers enrollment in this survey from different countries, except participants from Qatar (n=400, 38.8%) and India (n=161, 15.6%)

**Figure 1s:** Frequency (%) of healthcare workers enrollment in this survey from different countries, except participants from Qatar (n=400, 38.8%) and India (n=161, 15.6%), according to HCWs who worked in COVID-19 area or not
